# Characteristics and Clinical Implication of *UGT1A1* Heterozygous Mutation in Tumor

**DOI:** 10.3779/j.issn.1009-3419.2022.101.07

**Published:** 2022-03-20

**Authors:** Qian LI, Tao SUN, Hua ZHANG, Wei LIU, Yu XIAO, Hongqi SUN, Wencheng YIN, Yanhong YAO, Yangchun GU, Yan'e LIU, Fumei YI, Qiqi WANG, Jinyu YU, Baoshan CAO, Li LIANG

**Affiliations:** 1 Department of Medical Oncology and Radiation Sickness, Peking University Third Hospital, Beijing 100191, China; 2 Department of General Surgery, Peking University Third Hospital, Beijing 100191, China; 3 Research Center of Clinical Epidemiology, Peking University Third Hospital, Beijing 100191, China; 4 Department of Pharmacy, Peking University Third Hospital, Beijing 100191, China

**Keywords:** UGT1A1, Heterozygous mutation, Adverse reaction

## Abstract

**Background:**

The literature recommends that reduced dosage of CPT-11 should be applied in patients with *UGT1A1* homozygous mutations, but the impact of *UGT1A1* heterozygous mutations on the adverse reactions of CPT-11 is still not fully clear.

**Methods:**

A total of 107 patients with *UGT1A1* heterozygous mutation or wild-type, who were treated with CPT-11 from January 2018 to September 2021 in Peking University Third Hospital, were retrospectively enrolled. The adverse reaction spectra of patients with UGT1A1*6 and UGT1A1*28 mutations were analyzed. Adverse reactions were evaluated according to National Cancer Institute Common Terminology Criteria for Adverse Events (NCI-CTCAE) 5.0. The efficacy was evaluated according to Response Evaluation Criteria in Solid Tumors (RECIST) 1.1. The genotypes of UGT1A1*6 and UGT1A1*28 were detected by digital fluorescence molecular hybridization.

**Results:**

There were 43 patients with UGT1A1*6 heterozygous mutation, 26 patients with UGT1A1*28 heterozygous mutation, 8 patients with UGT1A1*6 and UGT1A1*28 double heterozygous mutations, 61 patients with heterozygous mutation at any gene locus of UGT1A1*6 and UGT1A1*28. *Logistic* regression analysis showed that the presence or absence of vomiting (*P*=0.013) and mucositis (*P*=0.005) was significantly correlated with heterozygous mutation of UGT1A1*28, and the severity of vomiting (*P* < 0.001) and neutropenia (*P*=0.021) were significantly correlated with heterozygous mutation of UGT1A1*6. In colorectal cancer, UGT1A1*6 was significantly correlated to diarrhea (*P*=0.005), and the other adverse reactions spectrum was similar to that of the whole patient cohort, and efficacy and prognosis were similar between patients with different genotypes and patients treated with reduced CPT-11 dosage or not.

**Conclusion:**

In clinical use, heterozygous mutations of UGT1A1*6 and UGT1A1*28 are related to the risk and severity of vomiting, diarrhea, neutropenia and mucositis in patients with Pan-tumor and colorectal cancer post CPT-11 therpy. In colorectal cancer, UGT1A1*6 is significantly related to diarrhea post CPT-11 use, efficacy and prognosis is not affected by various genotypes or CPT-11 dosage reduction.

## Introduction

There are more than 50 kinds of polymorphisms in the *UGT1A1* gene loci. Among them, the relationship between UGT1A1*28 and UGT1A1*6 gene polymorphisms and CPT-11 chemotherapy-related adverse reactions and efficacy is of particular concern^[1]^. SN-38 is the active metabolite of CPT-11. Studies have shown that some *UGT1A1* genotypes might affect the pharmacokinetics of SN-38 and therefore relate to CPT-11 induced drug toxicity reactions^[2]^.

Previous studies have found that at the same dose of CPT-11, patients with genetic mutations of UGT1A1*28 and UGT1A1*6 might face increased risk of neutropenia and diarrhea, especially in patients with homozygous mutations of UGT1A1*28 and UGT1A1*6, and in that case the dose of CPT-11 needs to be reduced. It has been reported that the gene activity will be reduced up to 70% by homozygous mutation of *UGT1A1*, and up to 30% by heterozygous mutation of *UGT1A1*. The dose reduction standard for CPT-11 is about 25%-30% in patients with homozygous mutation of *UGT1A1*^[3, 4]^. Patients with heterozygous mutations of *UGT1A1* were reported to have fewer adverse effects to CPT-11 than patients with homozygous mutations, but the incidence of adverse effects post CPT-11 were still higher than in wild-type patients^[5]^. Moreover, it was reported that the adverse effects to CPT-11 differed among patients with heterozygous mutations of *UGT1A1*^[6-8]^, and in patients with heterozygous mutation of *UGT1A1* at different gene loci^[9, 10]^. Thus, it remains elusive whether dose reduction of CPT-11 should be applied to patients with heterozygous mutation of *UGT1A1*^[11-13]^, and if dose reduction of CPT-11 would affect the efficacy and prognosis in these patients^[14]^.

Therefore, this study was designed to observe the difference in the adverse reaction spectrum to CPT-11 in panneoplastic and colorectal cancer patients with heterozygous mutation of *UGT1A1* and the impact of dose reduction of CPT-11 on efficacy and prognosis in these patients.

## Materials and methods

### Patient cohort

A total of 107 patients who were treated with CPT-11 in the Peking University Third Hospital from January 2018 to September 2021, and underwent *UGT1A1* genotyping screen before CPT-11, were enrolled. The inclusion criteria were: (1) Malignant tumors diagnosed by pathology, including colorectal cancer, lung cancer, gastric cancer and cholangiopancreatic cancer; (2) Eastern Cooperative Oncology Group (ECOG) performance status (PS) of 0-2; (3) The expected survival was more than three months with measurable lesions; (4) Aged between 18 and 80 years old; (5) No severe hepatic and renal insufficiency; (6) UGT1A1*6 or UGT1A1*28 heterozygous mutations or wild-type. The specific chemotherapy protocols were FOLFIRI/xeliri/CPT-11 for colorectal cancer, IP/CPT-11 for lung cancer, CPT-11 single drug±Apatinib for gastric cancer and CPT-11 for cholangiopancreatic cancer. The standard dose of the various protocols was decided according to National Comprehensive Cancer Network (NCCN) and Chinese Society of Clinical Oncology (CSCO) guidelines. The weekly dose intensity of CPT-11 did not exceed 90 mg/m^2^. For patients with serious adverse reactions or an initial ECOG score of 2 after the first cycle of chemotherapy, 10%-20% dose reduction of CPT-11 was applied. The exclusion criteria included infection, uncontrolled cardiovascular and cerebrovascular diseases, chronic diarrhea, bone marrow metastasis, lactation and pregnancy, UGT1A1*6/*28 homozygous mutations and double primary carcinoma. Patients were followed up by telephone call or clinical visit until October 2021. The study protocol passed the ethical review of the Peking University Third Hospital.

### Efficacy and toxicity assessment

Data including ECOG score, hyperbilirubinemia, smoking, gender, age, tumor type, number of treatment lines, treatment plan, CPT-11 combination or single drug, CPT-11 dose reduction and the amount of dose reduction of CPT-11 were obtained. Related adverse reactions included vomiting, mucositis, neutropenia, diarrhea, anemia, thrombocytopenia and fatigue were analyzed. Adverse reactions were evaluated according to National Cancer Institute Common Terminology Criteria for Adverse Events (NCI-CTCAE) 5.0. The efficacy was evaluated according to the Response Evaluation Criteria in Solid Tumors (RECIST) 1.1. Prognostic indicators include progression-free survival (PFS), which is defined as the time from the beginning of treatment to tumor progression or death. Overall survival (OS) is defined as the time from diagnosis to death due to any cause.

### Genotyping of UGT1A1*6 and UGT1A1*28

Whole blood samples were collected using ethylenediamine tetraacetic acid (EDTA) as an anticoagulant. Then, 200 μL of the blood sample was transferred to an Eppendorf (EP) tube, and ammonium chloride solution (Beijing Huaxia Times Gene Technology Development Co., Ltd.) for blood sample pretreatment for 5 min. The pretreated specimens were centrifuged at 700 × *g* for 5 min to obtain precipitated blood cells. Genomic DNA was extracted directly from the above precipitated blood cells using a nucleic acid purification kit (Beijing Huaxia Times Gene Technology Development Co., Ltd.) and vibrated for 1 min. After standing for 15 min, the universal kit for sequencing the reaction of a single gene locus (Beijing Huaxia Times Gene Technology Development Co., Ltd.) was used to detect the *UGT1A1* genotype (UGT1A1*6 and UGT1A1*28 gene polymorphisms).

### Data statistics

*Hardy-Weinberg* equilibrium test was used for genetic epidemiology. For continuous (quantitative) data, the *Shapiro* normality test was used to determine the normality of the sample data. If it conformed to a normal distribution, it was expressed as the Mean±SD. Student's *t* tests was used for comparisons between the two groups. If the data did not conform to a normal distribution, they were expressed as the median (25% quantile, 75% quantile), and *Wilcoxon* test was used for the comparisons between the two groups. Classified (qualitative) data were statistically described by frequency (percentage) and compared by *χ*^2^ test or *Fisher's* exact test. In the multivariate analysis of the association between adverse reactions and genotype mutations, GLM function was used for binary *Logistic* regression analysis. Each adverse reaction was taken as the dependent variable, and UGT1A1*6 mutation, UGT1A1*28 mutation, number of treatment lines, treatment regimen, smoking, gender, tumor type and hyperbilirubinemia were taken as the independent variables. When the independent variables were categorical classification variables, the minimum value was taken as the reference. When the independent variables were continuous variables, the continuous variables were directly included in the binary *Logistic* regression model. Two-tailed *P* < 0.05 was considered to be statistically significant.

## Results

There were 66 cases of colorectal cancer, 16 cases of lung cancer, 15 cases of biliary and pancreatic tumors and 10 cases of gastric cancer in this cohort. There were 43 patients with UGT1A1*6 heterozygous mutation, 26 patients with UGT1A1*28 heterozygous mutation, 8 patients with UGT1A1*6 and UGT1A1*28 double heterozygous mutations, 61 patients with heterozygous mutation at any gene locus of UGT1A1*6 and UGT1A1*28. There were 10 patients with wild-type, 41 with heterozygous mutation and 56 homozygous mutation of *SLCO1B1* gene. There were 46 patients receiving the first-line, 42 patients receiving the second-line, and 19 patients receiving the ≥third-line chemotherapy in this cohort. There were 85 patients treated with CPT-11 combined regimen and 22 patients with single CPT-1 regimen (Tab 1).

**Table 1:** Baseline characteristics of UGT1A1*6 and UGT1A1*28

Characteristics	UGT1A1*6		*P*	UGT1A1*28		*P*
	WT (*n*=64)	HM (*n*=43)		WT (*n*=81)	HM (*n*=26)	
Gender			0.124			0.129
Male	29 (45.31%)	26 (60.47%)		45 (55.56%)	10 (38.46%)	
Female	35 (54.69%)	17 (39.53%)		36 (44.44%)	16 (61.54%)	
Smoking history			0.587			0.489
Yes	22 (34.38%)	17 (39.53%)		31 (38.27%)	8 (30.77%)	
No	42 (65.62%)	26 (60.47%)		50 (61.73%)	18 (69.23%)	
Hyperbilirubinemia			< 0.001			0.060
Yes	4 (6.25%)	14 (32.56%)		10 (12.35%)	8 (30.77%)	
No	60 (93.75%)	29 (67.44%)		71 (87.65%)	18 (69.23%)	
SLCO1B1			0.742			0.260
WT	5 (7.81%)	5 (11.63%)		6 (7.41%)	4 (15.38%)	
HM	24 (37.50%)	17 (39.53%)		34 (41.98%)	7 (26.92%)	
M	35 (54.69%)	21 (48.84%)		41 (50.62%)	15 (57.69%)	
CRC			0.549			0.986
Yes	38 (59.38%)	28 (65.12%)		50 (61.73%)	16 (61.54%)	
No	26 (40.62%)	15 (34.88%)		31 (38.27%)	10 (38.46%)	
Treatment lines			0.830			0.635
First-line	26 (40.62%)	20 (46.51%)		34 (41.98%)	12 (46.15%)	
Second-line	26 (40.62%)	16 (37.21%)		31 (38.27%)	11 (42.31%)	
Third-line	12 (18.75%)	7 (16.28%)		16 (19.75%)	3 (11.54%)	
Regimen			0.682			0.191
Single	14 (21.88%)	8 (18.60%)		19 (23.46%)	3 (11.54%)	
Combined	50 (78.12%)	35 (81.40%)		62 (76.54%)	23 (88.46%)	
Dose decrease			0.043			0.619
≤10%	49 (76.56%)	25 (58.14%)		55 (67.90%)	19 (73.08%)	
> 10%	15 (23.44%)	18 (41.86%)		26 (32.10%)	7 (26.92%)	
Dose decrease			0.003			0.321
Yes	35 (54.69%)	11 (25.58%)		37 (45.68%)	9 (34.62%)	
No	29 (45.31%)	32 (74.42%)		44 (54.32%)	17 (65.38%)	
*WT: wild-type; HM: heterozygous mutation; M: homozygous mutation; CRC: colorectal cancer.						

### Adverse reaction spectrum and baseline characteristics of patients with heterozygous mutation of UGT1A1*6 and UGT1A1*28

In this study, the heterozygous mutation rates of UGT1A1*6 and UGT1A1*28 were 40.2% (43/107) and 24.3% (26/107), respectively. Results showed that heterozygous mutation of these two genes were significantly correlated with the occurrence and severity of multiple common clinical adverse reactions post CPT-11 treatment, such as vomiting, neutropenia, diarrhea and mucositis (Tab 2). Multivariate regression analysis showed that heterozygous mutation of these two genes were related to vomiting, diarrhea, neutropenia and mucositis post CPT-11 use (Tab 3).

**Table 2:** Adverse reaction spectrum of UGT1A1*6 and UGT1A1*28

Adverse reaction	UGT1A1*6		*P*	UGT1A1*28		*P*
	WT (*n*=64)	HM (*n*=43)		WT (*n*=81)	HM (*n*=26)	
Vomit grade			0.020			0.013
0	30 (46.88%)	16 (37.21%)		41 (50.62%)	5 (19.23%)	
1-2	25 (39.06%)	11 (25.58%)		25 (30.86%)	11 (42.31%)	
3-4	9 (14.06%)	16 (37.21%)		15 (18.52%)	10 (38.46%)	
Mucositis			0.322			0.005
Yes	46 (71.88%)	27 (62.79%)		61 (75.31%)	12 (46.15%)	
No	18 (28.12%)	16 (37.21%)		20 (24.69%)	14 (53.85%)	
Diarrhea grade			0.027			0.382
0-2	50 (78.12%)	25 (58.14%)		55 (67.90%)	20 (76.92%)	
3-4	14 (21.88%)	18 (41.86%)		26 (32.10%)	6 (23.08%)	
Neutropenia grade			0.005			0.241
0-2	43 (67.19%)	17 (39.53%)		48 (59.26%)	12 (46.15%)	
3-4	21 (32.81%)	26 (60.47%)		33 (40.74%)	14 (53.85%)	
Anemia			0.654			0.820
Yes	53 (82.81%)	37 (86.05%)		69 (85.19%)	21 (80.77%)	
No	11 (17.19%)	6 (13.95%)		12 (14.81%)	5 (19.23%)	
Thrombocytopenia			0.481			0.510
Yes	54 (84.38%)	34 (79.07%)		65 (80.25%)	23 (88.46%)	
No	10 (15.62%)	9 (20.93%)		16 (19.75%)	3 (11.54%)	
Fatigue			0.654			0.697
Yes	53 (82.81%)	37 (86.05%)		67 (82.72%)	23 (88.46%)	
No	11 (17.19%)	6 (13.95%)		14 (17.28%)	3 (11.54%)	

**Table 3:** *Logistic* regression of common adverse reactions

Adverse events	Gene type	*B*	*SE*	*Z*	*P*	OR (95%CI)
*Vomit	UGT1A1*6	0.524	0.438	1.198	0.231	1.69 (0.72-3.99)
	UGT1A1*28	1.491	0.568	2.623	0.009	4.44 (1.46-13.52)
*Vomit grade	UGT1A1*6	1.921	0.573	3.353	0.001	6.83 (2.22-20.98)
	UGT1A1*28	1.081	0.577	1.874	0.061	2.95 (0.95-9.14)
*Diarrhea	UGT1A1*6	0.128	0.453	0.283	0.777	1.14 (0.47-2.76)
	UGT1A1*28	-0.474	0.499	-0.949	0.343	0.62 (0.23-1.66)
*Mucositis	UGT1A1*6	0.601	0.447	1.346	0.178	1.82 (0.76-4.38)
	UGT1A1*28	1.371	0.484	2.831	0.005	3.94 (1.52-10.17)
*Neutropenia grade	UGT1A1*6	1.057	0.459	2.303	0.021	2.88 (1.17-7.08)
	UGT1A1*28	0.575	0.506	1.136	0.256	1.78 (0.66-4.79)
*After adjusting the number of treatment lines, treatment plan, smoking, gender, tumor type and hyperbilirubinemia.						

According to the statistical results of the above baseline tables, the UGT1A1*6 mutation is related to hyperbilirubinemia, the amount of CPT-11 dose reduction and whether dose reduction of CPT-11 took place, the degree of vomiting, the severity of diarrhea and neutropenia. These results show that the UGT1A1*6 mutation may be more likely to cause vomiting, diarrhea and neutropenia, which can be used as an important index to guide the CPT-11 dose reduction. At the same time, patients with UGT1A1*6 mutations are also more prone to concurrent hyperbilirubinemia. UGT1A1*28 is related to the severity of vomiting and mucositis.

### Multivariate analysis of common adverse reactions in patients with heterozygous mutation of UGT1A1*6 and UGT1A1*28

Multifactorial regression analysis showed that after adjusting the cofounders, UGT1A1*28 remained as an independent risk factor for vomiting and mucositis, and UGT1A1*6 was the independent risk factor for the severity of vomiting and degree of neutropenia (Tab 3).

### Analysis of adverse reactions and prognosis in colorectal cancer in patients with heterozygous mutation of UGT1A1*6 and UGT1A1*28

To eliminate the confounding factors caused by the difference in tumor species, the researchers further analyzed the features of patients with single colorectal cancer disease, which accounted for the highest proportion in this cohort. After adjusting the six variables including treatment line, treatment regimen, smoking, gender, tumor type and hyperbilirubinemia, multivariate *Logistic* regression analysis showed that UGT1A1*6 was significantly correlated with the presence or absence of vomiting, the severity of vomiting and diarrhea, and UGT1A1*28 was significantly correlated with the severity of vomiting (Tab 4). At the same time, multivariate *Cox* regression analysis of prognosis and survival of PFS (Tab 5) and OS (Tab 6) in colorectal cancer was carried out. Results showed that genotype, CPT-11 reduction degree or other factors did not affect prognosis and PFS in this patient cohort.

**Table 4:** *Logistic* regression analysis of adverse reactions in patients with colorectal cancer

Adverse events	Gene type	*B*	*SE*	*Z*	*P*	OR (95%CI)
*Vomit	UGT1A1*6	1.250	0.590	2.118	0.034	3.49 (1.10-11.10)
	UGT1A1*28	1.029	0.680	1.513	0.130	2.80 (0.74-10.62)
*Vomit grade	UGT1A1*6	2.236	0.786	2.843	0.004	9.36 (2.00-43.70)
	UGT1A1*28	1.481	0.761	1.945	0.052	4.40 (0.99-19.55)
*Diarrhea grade	UGT1A1*6	2.166	0.767	2.824	0.005	8.72 (1.94-39.22)
	UGT1A1*28	-0.899	0.963	-0.933	0.351	0.41 (0.06-2.69)
*Neutropenia grade	UGT1A1*6	1.484	0.595	2.492	0.013	4.41 (1.37-14.16)
	UGT1A1*28	0.825	0.668	1.235	0.217	2.28 (0.62-8.44)
*After adjusting the number of treatment lines, treatment plan, smoking, gender, tumor type and hyperbilirubinemia.						

**Table 5:** Results of *Cox* regression multivariate analysis of progression-free survival

Independent variable	*B*	*SE*	*Z*	*P*	HR (95%CI)
Gender	0.132	0.433	0.304	0.761	1.14 (0.49-2.67)
Hyperbilirubinemia	0.137	0.719	0.190	0.849	1.15 (0.28-4.69)
Complication	-0.581	0.537	-1.082	0.279	0.56 (0.20-1.60)
Metastasis	0.064	0.550	0.117	0.907	1.07 (0.36-3.13)
Regimen					
CPT-11	Ref.				
FOLFIR1	-1.238	0.689	-1.796	0.073	0.29 (0.08-1.12)
XELIRI	-0.599	0.671	-0.893	0.372	0.55 (0.15-2.05)
Age	-0.576	0.468	-1.232	0.218	0.56 (0.22-1.41)
UGT1A1*6	0.209	0.514	0.408	0.683	1.23 (0.45-3.37)
UGT1A1*28	0.724	0.521	1.391	0.164	2.06 (0.74-5.72)
First-line	1.118	0.657	1.702	0.089	3.06 (0.84-11.80)
≥Second-line	1.665	1.299	1.282	0.200	5.29 (0.41-67.70)
Dose decrease	-0.556	0.629	-0.884	0.377	0.57 (0.17-1.97)

**Table 6:** Multivariate analysis results of Cox regression for overall survival

Independent variable	*B*	*SE*	*Z*	*P*	HR (95%CI)
Gender	-1.495	1.252	-1.194	0.232	0.22 (0.02-2.61)
Hyperbilirubinemia	0.371	1.356	0.274	0.784	1.45 (0.10-20.66)
Complication	2.529	2.334	1.084	0.279	12.54 (0.13-1, 216.22)
Metastasis	3.008	1.767	1.702	0.089	20.24 (0.63-646.48)
Regimen					
CPT-11	Ref.				
FOLFIR1	-3.384	1.645	-2.057	0.040	0.03 (0.00-0.85)
XELIRI	-3.252	1.577	-2.062	0.039	0.04 (0.00-0.85)
Age	-1.284	1.309	-0.981	0.326	0.28 (0.02-3.60)
UGT1A1*6	-0.942	1.261	-0.747	0.455	0.39 (0.03-4.61)
UGT1A1*28	1.058	1.201	0.881	0.378	2.88 (0.27-30.29)
Dose decrease	0.441	1.938	0.228	0.820	1.55 (0.03-69.37)

## Discussion

CPT-11 has been approved as a first-line or second-line drug for colorectal cancer, lung cancer, pancreatic cancer, esophageal cancer, gastric cancer and other tumor types. Common adverse reactions include neutropenia and diarrhea. It can lead to interruption or cessation of the treatment, thereby jeopardizing the patient's prognosis and quality of life. Most of the metabolites of CPT-11, SN-38, are detoxified by uridine diphosphate-glucuronyl transferase (mainly *UGT1A1*). In patients with *UGT1A1* mutations, the detoxification process slows down, and adverse reactions might increase. Therefore, *UGT1A1* has been increasingly used as a reference target for dosage formulations in clinical use^[15]^. Many previous clinical studies regarding the clinical use of CPT-11 came from European and American patients, but results from Asian patients are scarce. It is known that there is a significant difference in the epidemiological distribution of UGT1A1*6 and UGT1A1*28 between Asians and Caucasians. The UGT1A1*28 polymorphism in the Caucasian population accounts for 30%-45%, and among East Asians, it accounts for only 10%-20%. Among Asians, the UGT1A1*6 polymorphism accounts for 16%-40%^[16]^. Therefore, more data from Asian patients are needed to better guide the clinical use of CPT-11 in Asian patients.

In this study, patients with heterozygous mutations in *UGT1A1* and treated with CPT-11 were included to explore the relationship between genotype and the characteristics of adverse reactions in these patients. We mainly found four points: First, the population in this cohort were all patients with stage Ⅲ inoperable or stage Ⅳ cancer. Results showed that UGT1A1*6 mutations are related to hyperbilirubinemia, how much and whether to reduce the dose, the degree of vomiting, the severity of diarrhea, and the severity of neutropenia, which shows that the patients with UGT1A1*6 heterogeneous mutation may be more likely to cause severe vomiting, diarrhea, and neutropenia, as well as hyperbilirubinemia. Thus, like for UGT1A1*6 homogenous patients, patients with UGT1A1*6 heterogeneous mutation might also undergoing dose reduction of CPT-11. UGT1A1*6 heterogeneous mutation can be used as an important indicator of decision making on dose reduction of CPT-11 and how much should be reduced. UGT1A1*28 heterogeneous mutation is also related to the severity of vomiting and mucositis. Second, multivariate *Logistic* regression analysis showed that the presence or absence of vomiting and mucositis was more related to the UGT1A1*28 heterogeneous mutation, and severe vomiting and severe neutropenia were more common in patients with heterogeneous mutation of UGT1A1*6. Third, to further clarify whether there is a difference in the adverse reaction spectrum between colorectal cancer and panneoplastic species, we performed multivariate *Logistic* regression analysis on 107 panneoplastic patients and 66 patients with simple colorectal cancer after adjusting for multiple variables. We found that the overall adverse reaction spectrum was similar in pantumor species and colorectal cancer, while in colorectal cancer, the UGT1A1*6 mutation is highly correlated with the severity of diarrhea, and in pantumor species, among them, UGT1A1*28 is significantly related to mucositis. Fourth, the efficacy and prognosis of mutant patients were similar from those of wild-type patients in colorectal cancer.

In terms of the types of adverse reactions, there have been numerous reports in the literature about the increased risk of diarrhea and neutropeniain patients with gene mutations. Previous sudies have found that diarrhea was significantly associated with UGT1A1*28 homozygous mutations^[17-19]^, in the high/medium dose group (> 125 mg/m^2^) of CPT-11, but not in the low-dose (< 125 mg/m^2^) group^[20]^. A study evaluating the combination of irinotecan and platinum in the treatment of colorectal cancer reported an increased risk of severe diarrhea in patients with UGT1A1*6 alleles^[21]^. Among white people, risk of neutropenia was high in patients with UGT1A1*28 genotype mutations^[22]^. In Asians, UGT1A1*6 alleles are reported to be associated with severe hematological toxicity^[23]^. Studies also demonstrated that in Asian cancer patients, the combination of UGT1A1*6 and UGT1A1*28 polymorphisms was associated with an increased risk of IRI-induced neutropenia^[6]^. A *meta*-analysis showed that Asians with lung cancer and UGT1A1*6 genetic polymorphism faced a higher risk of neutropenia and diarrhea after IRI chemotherapy. However, UGT1A1*28 is weakly correlated with diarrhea and neutropenia^[14]^. However, there are very few reports in the literature about the risk of mucositis and vomiting, and only a few studies are involved^[24]^. Our research found an increased risk of vomiting and mucositis, which may indicate that in patients with *UGT1A1* mutations, we should pay more attention to these two often overlooked adverse reactions related to CPT-11 use in daily clinical practice.

In this study, we also found that UGT1A1*6 is significantly correlated with the increased risk of bilirubin, which is mainly because the metabolism of bilirubin also depends on this gene. This is consistent with the literature report^[25]^. A number of previous studies have shown that homozygous UGT1A1*28 and homozygous UGT1A1*6 are associated with an increased risk of hyperbilirubinemia^[26, 27]^. It was shown that patients with total bilirubin levels between 1.0 mg/dL and 2.0 mg/dL faced higher riks of grade 3-4 neutropenia. Perhaps in the future, if there is no chance to detect *UGT1A1* genotype, clinicians can judge the potential risk of adverse reaction post CPT-11 use through elevating the bilirubin level, patients with elevated bilirubin should be monitored more cautiously during CPT-11 therapy.

Some studies suggest that only high doses of CPT-11 could induce severe adverse effects in patients with *UGT1A1* polymorphisms. Especially when the dose is ≥180 mg/m^2^, UGT1A1*28 is related to neutropenia and diarrhea. At normal doses, the alarming role of UGT1A1*6 on neutropenia and diarrhea is greater than UGT1A1*28^[28]^. This is consistent with our findings. Although high doses CPT-11 would certainly relate to a higher risk of adverse drug effects in patients with *UGT1A1* mutations, the achieved benefit might also greater. A study showed that patients with UGT1A1*1/*1 and UGT1A1*1/*28 genotypes could tolerate high doses of CPT-11 and more favorable objective response rate (ORR) was achieved without significant adverse events^[29]^. Another study found that the dose titration plan of CPT-11 is a satisfactory option for achieving favorable ORR and disease control rate (DCR). Metastatic colorectal cancer patients with the *UGT1A1* wild-type genotype can tolerate higher doses of CPT-11 and may obtain more favorable clinical results without significantly increased toxicity. Therefore, under the guidance of *UGT1A1* gene detection, use of CPT-11 could be optimized in daily clinical practice.

Most studies have failed to observe the correlation between *UGT1A1* genotype and prognosis^[30]^. However, some studies found that the prognosis is more favorable in patients with mutations. In a study on small cell lung cancer, it was shown that UGT1A1*6 and UGT1A1*28 polymorphisms were associated with increased gastrointestinal toxicity and improved OS, respectively^[31,32]^. In colorectal cancer, good prognosis was evidenced in patients with the UGT1A1*28 polymorphism^[33]^. The possible mechanism may be that patients with gene mutations had higher blood drug concentration at the first 1.5 h timepoint^[34]^, thereby achieving higher efficacy. However, there are some opposite findings in that prognosis is better in wild-type patients. For example, in the third-line treatment of gastric cancer, it was found that patients with wide type *UGT1A1* had better OS, DCR and time to progress (TTP) than mutant patients^[35]^. The possible mechanism may be that higher doses tolerated by these patients might be linked with the better prognosis.

In our study, no difference was found in prognosis between mutant and wild-type patients. The possible reasons might be as follows: (1) A better prognosis in colorectal cancer comes from the exploration of larger doses. In our research, we used conventional standard doses; (2) The types of tumors that suggest a better prognosis in conventional doses are mostly reflected in patients with nonintestinal cancer. This study mainly included patients with intestinal cancer, so there may be differences in tumor types; (3) In our clinical practice, most of the patients with mutations were forced to reduce the dose due to severe adverse reactions, but the prognosis was not affected in the end. This may be because the mutation itself is a protective factor for the prognosis, neutralizing the reduction. The unfavorable factors brought about, so overall no difference in prognosis was found; (4) There is a need for longer-term follow-up and a larger sample size.

Some people think that routine testing of CPT-11 for the gene before use will increase the economic burden of patients. However, from an economic perspective, testing the gene can save costs. Related pharmacoeconomic studies have shown that compared with no genotyping, genetic testing only leads to a marginal increase in quality-adjusted life years, but the cost per patient is reduced by US＄651.12 and US＄805.22, respectively^[36]^. One-way sensitivity analysis shows that the model is relatively robust. Therefore, *UGT1A1* genotyping saves costs for Chinese patients with colorectal cancer^[37]^.

Under the guidance of the *UGT1A1* test, more bold attempts have been made in the treatment dose and program. Innocenti *et al*.^[38]^ has reported that the dose of CPT-11 can be individualized according to the differential expression of the UGT1A1*28 genotype. McLeod *et al*.^[39]^ found that different dose levels of CPT-11 and different combination schemes are closely related to pharmacogenomics predicting adverse reactions. Follow-up scholars have carried out a variety of explorations under the guidance of genes. For example, in the poor prognosis of *BRAF* mutant metastatic colorectal cancer, the dose of some patients could be increased under the guidance of genes to achieve a better curative effect^[40]^. Neoadjuvant chemoradiation for rectal cancer also uses genetic testing as a guide to improve pathological complete response (pCR), and the efficacy of high-dose patients is better^[41]^. In locally advanced gastroesophageal junction tumors, different dose gradient treatments are given according to the genotype, which improves the perioperative downstage rate and R0 resection rate^[42]^. Under the guidance of *UGT1A1* genotype, some researchers explored the efficacy and safety of the regimen of regorafenib combined with increased-dose FOLFIRI for metastatic colorectal cancer patients^[43]^. In gastrointestinal cancer, giving FOLFIRABRAX to patients who can tolerate it under the guidance of genotype has achieved better efficacy^[44]^. Therefore, *UGT1A1* detection can help us boldly explore more intensitive therapy for appropriate patients.

Although a variety of studies have found that *UGT1A1* affects adverse reactions, the adverse reaction spectrum and predictive value obtained from each study were different^[45]^. On the one hand, this may be due to different chemotherapy regimens (dose and cycle) and different tumor types. On the other hand, it may be that *UGT1A1* affects the metabolism of CPT-11. The plasma area under curve (AUC) of SN38 is related to many factors, including *ABCC*, *CYP3A*, *ABCB1* and *CES*^[46-48]^. Therefore, the risk of adverse reactions in our patients was largely dependent on and not only dependent on the *UGT1A1* gene. The risk and degree of adverse reactions are the result of the joint action of multiple metabolic genes. The clinical judgment of whether to reduce requires a comprehensive judgment based on the actual situation of the patient.

Based on our research results, we have the following tips for clinical practice. In addition to prophylactic leukogenics and antidiarrheal therapy, we also need to strengthen antiemetics. Try to give triple or even quadruple antiemetic programs. Patients with UGT1A1*28 mutations also have an increased chance of mucositis. More oral care, vitamin supplementation, *etc*. should be given to such patients to prevent adverse reactions to ensure that the dose completes the full course of chemotherapy. At the same time, patients with hyperbilirubinemia need to pay special attention. In patients whose *UGT1A1* has not been detected clinically, if bilirubin is elevated, the occurrence of adverse reactions can be carefully prevented, and the dose should be reduced if necessary.

This study also has several limitations, such as insufficient sample size, insufficient median follow-up time, and few cases of other tumor types except for colorectal cancer. This study is a retrospective study, and there are many factors of bias. For example, there are differences in the regimen, although studies have suggested that the combination of XELIRI and FOLFIRI has similar safety and effectiveness in different genotypes^[49]^, but there are still differences in other intraspecific research protocols. At the same time, due to the problem of sample size, it has not yet been possible to answer the reduction standard in high-risk groups. It's still not clear to determine the best range of reduction to ensure the best curative effect and minimal adverse reactions.

## Conclusion

UGT1A1*6 mutations and hyperbilirubinemia often occur concurrently. UGT1A1*6 heterozygous mutations will affect the amount of dose reduction and whether it is to be reduced. At the same time, it is related to the degree of vomiting, the severity of diarrhea, and the severity of neutropenia. UGT1A1*28 is related to the severity of vomiting and mucositis. In colorectal cancer, the adverse reaction spectrum is similar to pantumoma except UGT1A1*6, which is significantly related to the severity of diarrhea. Genotype, CPT-11 reduction degree or other factors did not affect prognosis and PFS in colorectal cancer patient cohort. Since *UGT1A1* is an important metabolic gene, it is not only detected in the peripheral blood of *UGT1A1*, but now studies are beginning to explore the distribution and role of this metabolic enzyme in organs and tissues^[49]^. There may be more exploratory studies on *UGT1A1* in the future.
